# Improved Tapaswini having four BB resistance genes pyramided with six genes/QTLs, resistance/tolerance to biotic and abiotic stresses in rice

**DOI:** 10.1038/s41598-018-20495-x

**Published:** 2018-02-05

**Authors:** Gitishree Das, Gundimeda J. N. Rao, M. Varier, A. Prakash, Dokku Prasad

**Affiliations:** 1 Crop Improvement Division, ICAR-National Rice Research Institute, Cuttack, Odisha 753006 India; 2 Research Institute of Biotechnology & Medical Converged Science, Dongguk University-Seoul, Ilsandong-gu, Gyeonggi-do 10326 Republic of Korea; 3 Department of Bio Sciences and Bio Engineering, Indian Institute of Technology Guwahati, Guwahati, Assam 781039 India; 4 NRRI-Central Rainfed Upland Rice Research Station, Hazaribagh, Jharkhand 825301 India; 5 Crop Protection Division, ICAR-National Rice Research Institute, Cuttack, Odisha 753006 India; 6 Kaveri Seeds, Secunderabad, Telangana 500003 India

## Abstract

Rice, a major food crop, is grown in a wide range of ecological conditions and suffers significant yield losses as it is constantly exposed to a wide range of environmental and biotic stresses. The prevalence of different biotypes/strains has necessitated assembling of numerous resistance genes/QTLs into elite genotypes to confer a broader scale of resistance. The current study reports successful pyramiding of genes/QTLs that confer tolerance/resistance to submergence (*Sub1*), salinity (*Saltol*), blast (*Pi2*, *Pi9*) and gall midge (*Gm1*, *Gm4*) to supplement the four bacterial blight resistance genes (*Xa 4*, *xa5*, *xa13*, *Xa21*) present in Improved Tapaswini, an elite cultivar. The precise transfer of genes/QTLs was accomplished through effective foreground selection and suitable gene pyramids were identified. Background selection was practiced using morphological and grain quality traits to enhance the recovery of the recurrent parental genome. In the bioassays, the pyramids exhibited higher levels of resistance/ tolerance against the target stresses. The novel feature of the study was successful pyramidization and demonstration of the function of ten genes/QTLs in a new genotype. This success can stimulate several such studies to realize the full potential of molecular plant breeding as the foundation for rice improvement.

## Introduction

Rice (*Oryza sativa* L.), is the primary source of food for more than half of the world’s population^1^ and is being cultivated under a wide range of environments, from arid highlands to flooded lowlands, high humidity to high temperature climates and also in acid, saline and alkaline soils. The rice plant growth and productivity, exposed to a wide range of environmental stresses such as submergence and salinity, suffer significant crop losses globally year after year. A range of biotic factors, which can be insect pests such as rice gall midge or diseases like bacterial leaf blight and blast, severely affect rice grown throughout tropical, subtropical and temperate areas of Asia.

Bacterial blight (BB), caused by *Xanthomonas oryzae* pv. *oryzae* (Xoo), is one of the oldest and most devastating diseases of rice throughout the world^2^ and significant yield losses up to 80–100% from bacterial blight infection were known to occur^3^ and when infection occurs during panicle initiation or earlier, the grain development will also be severely affected. As chemical control of the disease is not effective and reliable, genetic enhancement of host plant resistance is a viable and practical option against bacterial blight^4^.

In rice, the genetics of resistance to the pathogen has been well characterized and till date, thirty nine BB resistance genes, series from *Xa1* to *Xa39* have been identified from diverse sources^5,6^ and of these, nine genes (*Xa1*, *Xa3/Xa26*, *xa5*, *xa13*, *Xa10*, *Xa21*, *Xa23*, *xa25* and *Xa27*) have been isolated and characterized^7,8^ while seven genes (*Xa4*, *Xa7*, *Xa22*, *Xa30*, *Xa31*, *Xa33*, and *xa34*) have been fine-mapped^9^.

Breakdown of resistance in varieties having a single resistance gene has been reported in rice^10–12^ after 2 or 3 years as a result of shifts in the frequency of pathotypes or the emergence of new ones through mutation or other mechanisms. Multiple resistance genes confer durable broad spectrum resistance through synergistic and complementary gene action to a wide range of races compared to one, two and three gene combinations^13–15^.

Among the several fungal diseases infecting the rice plant, the rice blast disease, caused by an ascomycete fungus *Magnaporthe grisea* Barr. (Telomorph *Pyricularia oryzae* Sacc), is one of the major destructive disease on rice. Also known as rice fever disease, it was reported in all the rice growing countries of the globe^16–18^ and it infects panicles, culm and leaves of the rice plants, consequently reducing photosynthetic efficiency and yield of rice grain^19,20^ and causes 40–70% loss of rice grain^21,22^. For management of blast disease, use of chemical pesticides is not considered as an ecologically sound approach and fortification of host plant was a highly reasonable method^22^.

So far, more than 100 blast resistance (R) genes and 350 QTLs associated with resistance to blast have been reported. Between them, 26 blast resistance genes (*Pib*, *Pita*, *Pi54*, *Pid2*, *Pi9*, *Pi2*, *Pizt*, *Pi36*, *Pi37*, *Pikm*, *Pi5*, *Pit*, *Pid3*, *pi21*, *Pish*, *Pb1*, *Pik*, *Pikp*, *Pia*, *Pi25*, *Pid3A4*, *Pi35*, *NLS1*, *Pikh*, *Pi54* and *Pi54rh*) have been cloned and functionally authenticated^22–30^. Stacking blast resistance genes into a single cultivar has been recommended approach to broaden the resistance spectrum of genotypes as well as to achieve strong resistance^17,22,31^.

Asian rice gall midge (*Orseolia oryzae*), a major insect pest of rice occur mainly in wet season, in specific provinces of central, south, and east India, resulting major yield loss, mostly throughout the kharif season. Seven biotypes of the gall midge have been reported on rice so far^32^. Genetic studies have identified 11 major genes conferring resistance to gall midge^33,34^. Of these, eight genes (*Gm1*, *Gm2*, *Gm4*, *Gm5*, *Gm6*, *Gm7*, *Gm8 and Gm11*) have been tagged and mapped^34,35^.

In the rainfed lowlands of South and South East Asia, submergence stress of rice is a foremost frequent problem for rice farmers^36^. Flooding is a foremost natural tragedy that has a disadvantageous consequence on growth and health of plant in natural and agricultural environment^37–39^. In submergence stress and later desubmergence, rice plants use to confront several external challenges sequentially, which produce diverse inner stress which influence the growth and survival of rice plant. In submergence stress, it significantly reduces the gas diffusion rate, restricts uptake of oxygen and ineffective anaerobic metabolism^40^. Muddy floodwaters decreases light accessibility and slows down underwater photosynthesis. Constraint of efficient gas exchange as well highly limits rate of transpiration^41^. Under long-standing submergence stress, due to these conditions rice plants gets energy hunger and deficiency of nutrient. As a result of which the plant starts decaying and dying^39^. The yield loss can reach nearly 70% due to submergence alone^42^. The average yield loss from submergence is estimated about 80 kg/ha^43^.

Submergence tolerance is an important trait for rice in rainfed lowland conditions and is being evaluated as a weed control strategy for rice seeded directly into standing water. The *indica* cultivar FR13A, is well known for its tolerance to submergence which can withstand up to 14 days of total submergence owing to submergence1 (*Sub1*), a major quantitative trait locus mapped close to the centromere of chromosome 9^15,44–48^. Around eight *Sub1* varieties were developed and in Asia through incorporation of *Sub1* from FR13A into elite varieties.

The most extensive soil problem in the world is salinity and it is estimated that over 150 million hectares of potential rice land is affected by salinity^49^ and is considered as one of important physical factor influencing rice production. Salt tolerance has evolved and there are naturally occurring salt tolerant trees (mangroves), shrubs, grasses and herbs and in general, none of the crop plants are able to tolerate even a quarter of seawater without yield loss. Rice plant is classified as a salt sensitive crop^50^ and it is comparatively tolerant of salt stress during germination, active tillering and towards maturity and is sensitive during early seedling and reproductive stages^51^.

Tolerance to salinity is complex, involving a number of different physiological mechanisms, such as sodium exclusion from roots, controlled sodium transport between root and shoot, and sequestering of sodium in older tissues and in the vacuoles. A few traditional *indica* rice varieties such as Nona Bokra, Pokkali and Kala-rata are salinity stress tolerant rice varieties^52^. Even though several genes likely to contribute salinity stress tolerance, but the most important QTL for salinity tolerance designated as *Saltol*, present on the short arm of chromosome 1^53^, confers seedling stage tolerance. The Saltol QTL was transferred into elite IRRI breeding lines and a breeding line FL478, contains a 1 Mb DNA fragment (10.6–11.5 Mb on chromosome1) that contains the entire Saltol locus of Pokkali and the fragment was flanked by IR29 alleles and the breeding line displays high levels of tolerance to salinity^54^.

Incidences of different biotypes/strains/stresses have necessitated assembling of numerous resistance/tolerance genes into background of high yielding cultivars to confer a broad scale of resistance. Enhancement of broad spectrum resistance/tolerance capabilities of a genotype can be achieved with precision through introgression of additional resistance genes/QTLs using marker-assisted backcrossing (MAB) approach^55^ and the same cannot be accomplished through conventional approaches even with many generations of backcrosses^56^. With the availability of tightly linked PCR based (STS) markers for target genes and a large number of markers for background selection^57^, the pyramiding of the target genes is possible in minimum number of backcrosses effectively with significant savings in time, space, labor and money. Successful transfer of genes/QTLs into agronomically superior cultivars has been reported earlier in rice but all the reports deals with pyramiding of genes/QTLs of one or two traits only. In this study, an attempt was made to pyramid ten genes/QTLs in Improved Tapaswini to fortify the host plant defenses against several biotic and abiotic stresses simultaneously.

## Results

### Parental polymorphism survey

**Figure 1:**
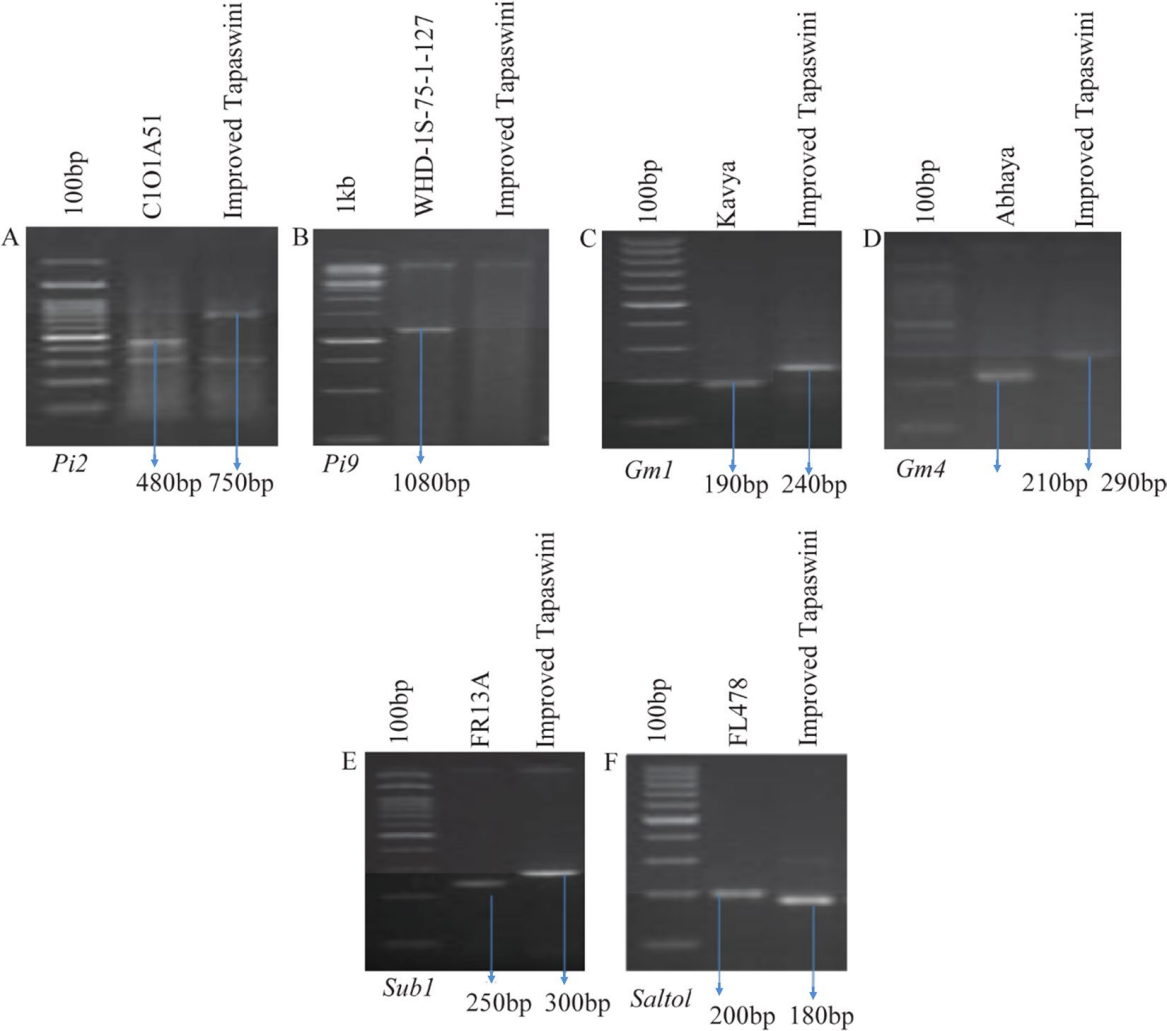
Parental polymorphism survey through PCR assays (cropped gels displayed). (**A**) Amplification with RG64 (digested with *HaeIII*) *linked to Pi2*. (**B**) Amplification with P28 linked to *Pi9*. (**C**) Amplification with RM444 linked to *Gm1*. (**D**) Amplification with RM547 linked to *Gm4*. (**E**) Amplification with SUB1BC2 linked to *Sub1*. (**F**) Amplification with RM10745 linked to *Saltol*. Arrows indicates amplicons linked to resistance and susceptible alleles.

Parental polymorphism survey revealed distinct polymorphism for the markers tested for the traits to be introgressed into Improved Tapaswini i.e. RG64 (C1O1A51-*Pi2*), P28 (WHD-1S-75-1-127- *Pi9*), RM444 (Kavya-*Gm1*), RM547 (Abhaya-*Gm4*), SUB1BC2 (FR13A-*Sub1*QTL) and RM10745 and (FL478-*Saltol* QTL) (Fig. 1 and Table 1). In the bioassays conducted against GM, blast and screenings against submergence and salinity, the respective donors showed high levels of resistance/tolerance for the stresses while Improved Tapaswini was susceptible for all the stresses tested. However, in the bioassays conducted against eight isolates of *Xoo*, Improved Tapaswini, the recurrent parent expressed high levels of resistance and the resistance levels are similar to the level expressed by IRBB 60, the line used as donor in developing Improved Tapaswini and both exhibited very short lesion lengths (data not shown).

**Table 1:** List of resistance/tolerance genes/QTLs, donors and markers employed in the study.

Genes/QTLs	Trait	Donors	Ch No.	Linked Markers	References
*Xa4*	BB Resistance	Improved Tapaswini	11	P1,P2	Ma *et al*.^57^
*xa5*	BB Resistance	Improved Tapaswini	5	RG556	Yoshimura *et al*.^101^
*xa13*	BB Resistance	Improved Tapaswini	8	Xa13prom	Singh *et al*.^102^
*Xa21*	BB Resistance	Improved Tapaswini	11	pTA 248	Ronald *et al*.^103^
*Pi2*	Blast Resistance	C1O1A51	6	RG64	Hittalmani *et al*.^66^
*Pi9*	Blast Resistance	WHD-1S-75-1-127	6	P28	Qu *et al*.^67^
*Gm1*	Gallmidge Resistance	Kavya	9	RM444	Biradar *et al*.^84^
*Gm4*	Gallmidge Resistance	Abhaya	8	RM547	Nair *et al*.^105^
*Sub1*	Submergence	FR13A	9	SUB1BC2	Xu *et al*.^106^
*Saltol*	Salinity	FL478	1	RM10745	Bonila *et al*.^53^

### Pyramiding of target genes/QTLs

**Figure 2:**
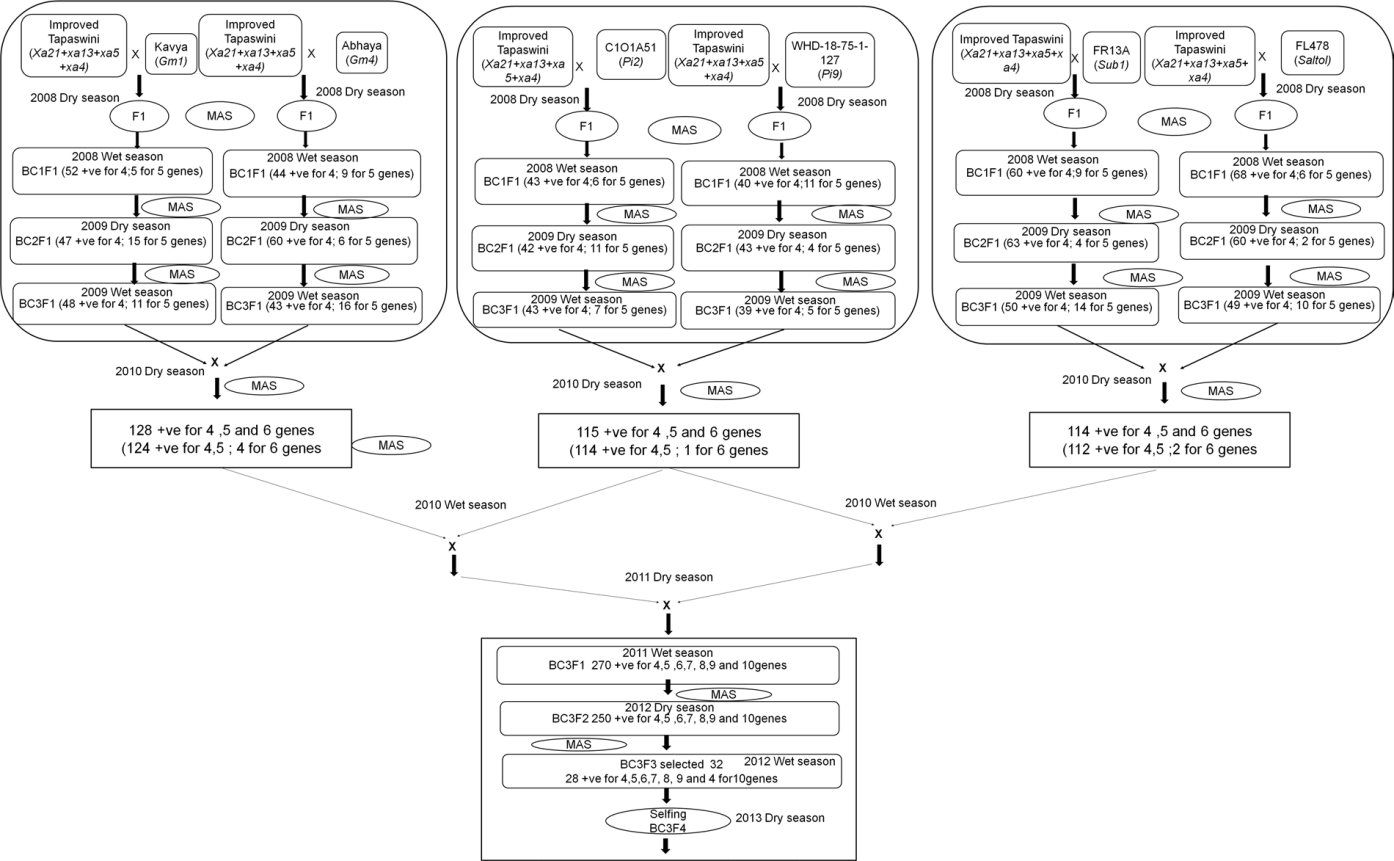
Flow diagram depicting the different steps of development of gene pyramids of Improved Tapaswini.

**Figure 3:**
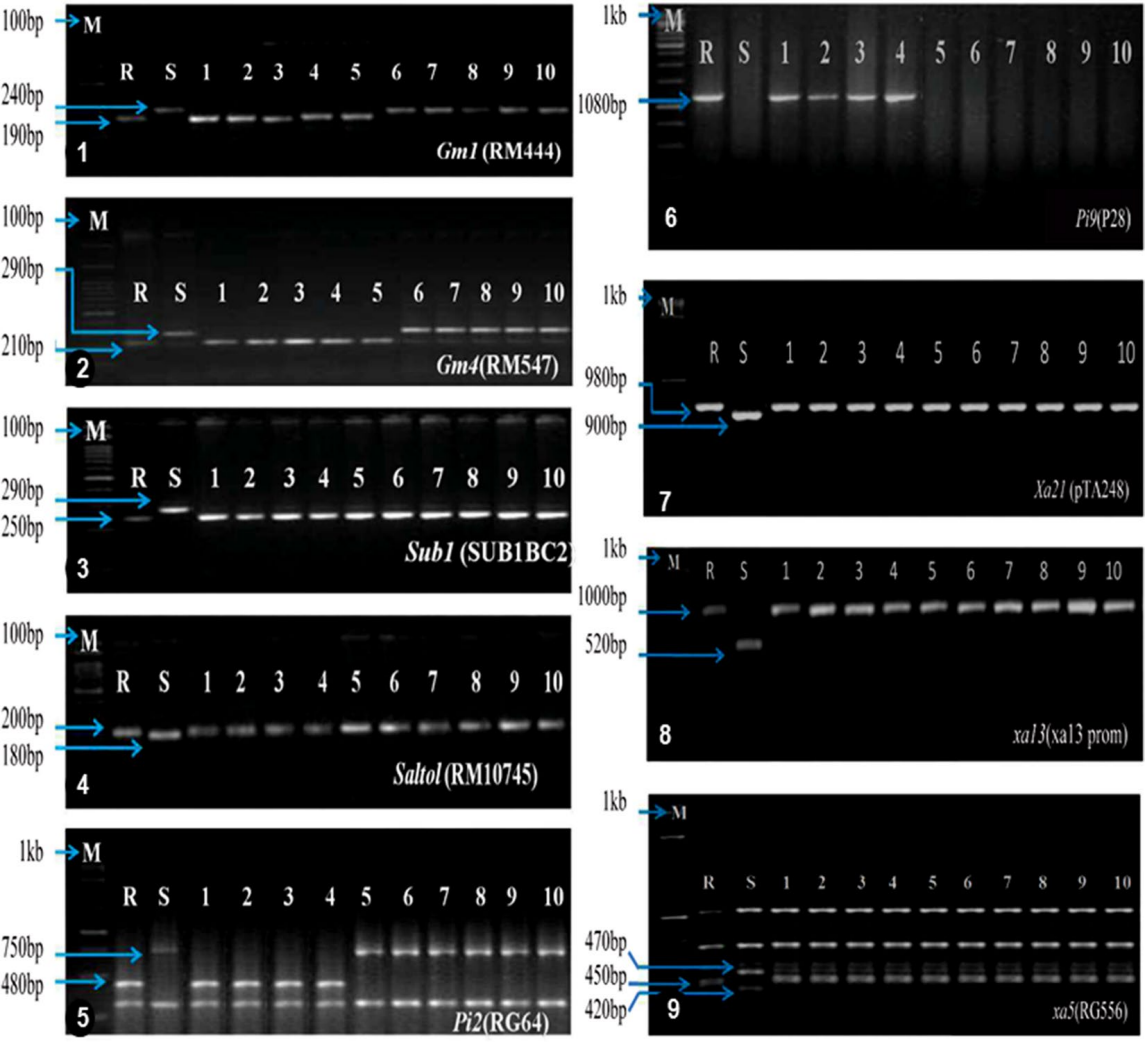
PCR analysis of gene pyramids for the presence target allele(s) (cropped gels displayed). 1. DNA amplification of *Gm1* alleles using the primer RM444. 2. DNA amplification of *Gm4* alleles using the primer RM547. 3. DNA amplification of *Sub1* allele using the primer SUB1BC2. 4. DNA amplification of *Saltol* alleles using the primer RM10745. 5. DNA amplification of *Pi2* alleles using the primer RG64 and digested with *HaeIII*. 6. DNA amplification of *Pi9* alleles using the primer P28. 7. DNA amplification of *Xa21* alleles using the primer pTA248. 8. DNA amplification of *xa13* alleles using the primer xa13Prom. 9. DNA amplification of *xa5* alleles using the primer RG556 and digested with *DraI*. M = Marker. For *Gm1*, *Gm4*, *Sub1* and *Saltol* the M.W. marker was100 bp ladder and for *Pi2*, *Pi9*, *Xa21*, *xa13* and *xa5* the M.W. marker was 1 kb ladder. S = Recurrent parent (susceptible) R = Resistant (donor) parent. The numbers (1–10) represent different gene pyramids. 1 = ITGP1, 2 = ITGP2, 3 = ITGP4, 4 = ITGP5, 5 = ITGP7, 6 = ITGP9, 7 = ITGP10, 8 = ITGP14, 9 = ITGP18, 10 = ITGP20.

The conventional backcross breeding method was practiced to transfer six new genes/QTLs to complement the four resistance genes present in Improved Tapaswini. In the first cycle, the recurrent parent was crossed with all the six donor parents separately and each of the F_1_s was backcrossed to the recurrent parent and each population was advanced till BC_3_F_1_ generation (Fig. 2). At each generation, marker assisted selection was carried out with the respective markers and plants having resistant alleles were selected. From BC_1_F_1_ onwards, stringent selection was also practiced for selecting plants similar to the recurrent parent in morphology along with forward selection for resistant alleles and only plants with desired morphology and alleles were advanced. At the end of the first cycle, six different back cross derivatives each having a desired allele of the target (six) trait(s) were generated. These six recombinants were used to develop three F_1_ hybrids, using two pyramids in each cross and both forward and background (morphology) selection was employed in the hybrids generated as described earlier. At the end of the second cycle, three recombinants, each with two resistance alleles of target genes/QTLs were developed. In the third cycle, the three recombinants were inter crossed to generate two different F_1_ hybrids as shown in Fig. 2 and after ensuring their hybrid nature, these two F_1_s were crossed again to generate a hybrid in the fourth cycle to pool all the genes/QTLs. A large number of hybrid plants were generated and from the population generated, after forward selection, two hundred and seventy plants (having resistance alleles of 4, 5, 6, 7, 8, 9, 10 genes/QTLs) and close similarity to recurrent parent in morphology were selected and carried forward (Fig. 2). From a large BC_3_F_2_ population, 250 lines having the resistance alleles in homozygous state were selected. From these lines, 32 lines with different gene combination were selected for different bioassays and characterization for morphological and quality traits in BC_3_F_3_ generation. Ten promising lines (Fig. 3) were selected for further experiments. Further refinement of gene pyramids is continuing.

One important observation was the presence of interaction between the BB genes. Of the different combinations possible, *Xa4* in all combinations was recovered, while the combinations with *xa5* with the other two genes i.e. *xa5* + *xa13* and *xa5* + *Xa21* were not recovered in the populations. Since the four gene (*Xa4* + *xa5* + *xa13* + *Xa21*) combination was observed to confer high levels of resistance, selection was practiced for the combination in all generations and lines homozygous for all the R alleles of all four genes were selected.

### Bioassays

#### Bacterial blight

**Figure 4:**
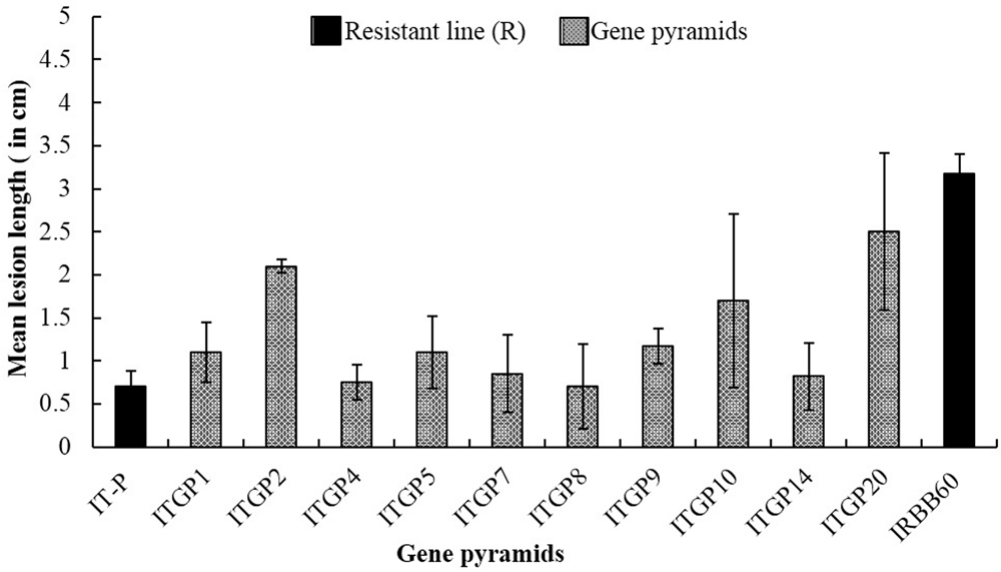
Disease reaction of different gene pyramids against bacterial blight.

In the bioassays against bacterial blight, the presence of high levels of resistance was confirmed in Improved Tapaswini, the recurrent parent. Among the tested pyramided lines, the lesion length was in the range of 0.70–2.50 cm while on IRBB 60, the resistant control, the length was ~3 cm (Fig. 4).

#### Rice gall midge

**Figure 5:**
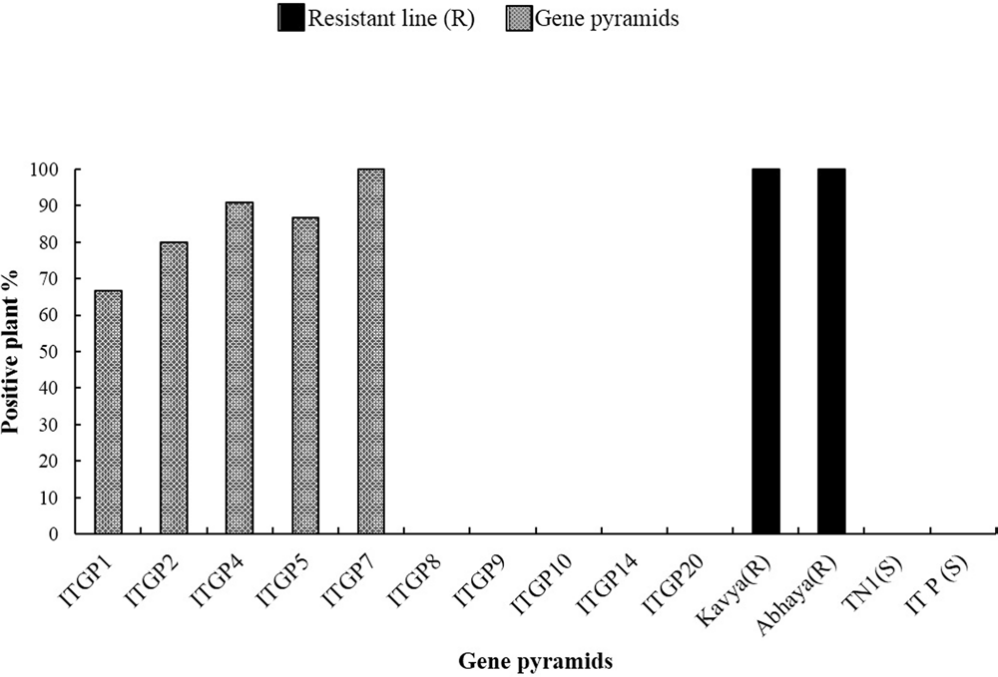
Disease reaction of different gene pyramids against gall midge.

The bioassays against gall midge were conducted employing Kavya (*Gm1*) and Abhaya (*Gm4*), the donors as resistant controls and T(N)1 and Improved Tapaswini as susceptible controls. The donors exhibited high levels of resistance and silver shoot formation was not observed in them. In susceptible controls [T(N)1; Improved Tapaswini], the incidence of galls/silver shoot was 100% suggesting complete susceptibility. Out of the ten gene pyramids (Fig. 3), ITGP7 (IT + *Gm1* + *Gm4*) showed high levels of resistance similar to resistant controls while in four gene pyramids (ITGP1, ITGP2, ITGP4 and ITGP5), the resistance levels are in the range of 66.6–90.9%. In five gene pyramids, the positive plants were below 30% (Fig. 5).

#### Blast

The screening against blast was carried out with C1O1A51 and WHD-1S-75-1-127 (donors) as resistant controls, and NSN1, HR12 and Improved Tapaswini as susceptible controls. In the bioassays, the resistant controls showed high levels of resistance and exhibited a resistant reaction (R) with a score ‘0’ whereas the recurrent parent exhibited a score of 4 similar to susceptible controls suggesting susceptible nature (S). The gene pyramids ITGP2 and ITGP5 (IT + *Pi2* + *Pi9*) exhibited a high degree of resistant reaction (R) with a score of 0 while ITGP1 and ITGP4 exhibited a moderately resistant reaction (MR) with a score 3. It is interesting to observe that ITGP8, despite having only *Pi2* also exhibited a moderately resistant reaction (MR) with a score 3. Five lines that were without any of the blast genes, exhibited a susceptible reaction with a score 4 (Table 2).

**Table 2:** Reaction of selected gene pyramids of Improved Tapaswini against blast.

Pyramid line	Disease score	Reaction
ITGP1	3	MR
ITGP2	0	R
ITGP4	3	MR
ITGP5	0	R
ITGP7	4	S
ITGP8	3	MR
ITGP9	4	S
ITGP10	4	S
ITGP14	4	S
ITGP20	4	S
Improved Tapaswini (P)	4	S
C1O1A51 (Donor)	0	R
WHD-1S-75-1-127 (Donor)	0	R
NSN1 (Susceptible control)	4	S
HR12 (Susceptible control)	4	S

ITGP 1- ITGP 20 = Improved Tapaswini gene pyramids.

#### Submergence

**Figure 6:**
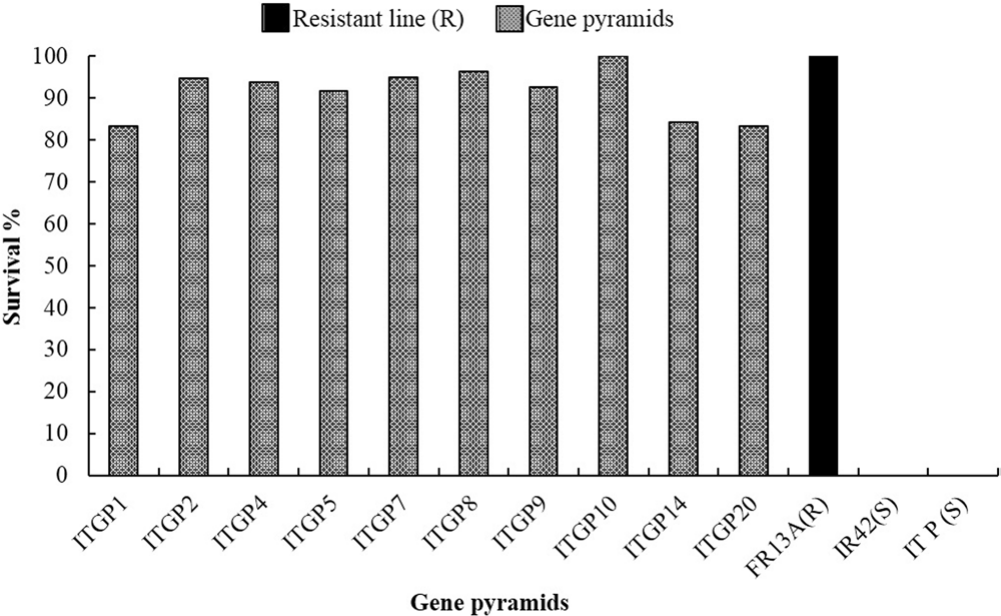
Survival rates of different after submergence stress and desubmergence.

The screening against submergence was conducted with FR13A, the donor as the resistant control, and IR42 and Improved Tapaswini as susceptible controls. The resistant control FR13A, showed 100% regeneration after 15 days of submergence stress and eight days of desubmergence while both the recurrent parent and IR42 completely died after the submergence stress. All the ten selected pyramid lines having *Sub1* QTL showed >83% survival. One gene pyramid, ITGP10, exhibited complete survival (100%) similar to resistant control while six pyramid lines showed >91% survival (Fig. 6).

#### Salinity

**Figure 7:**
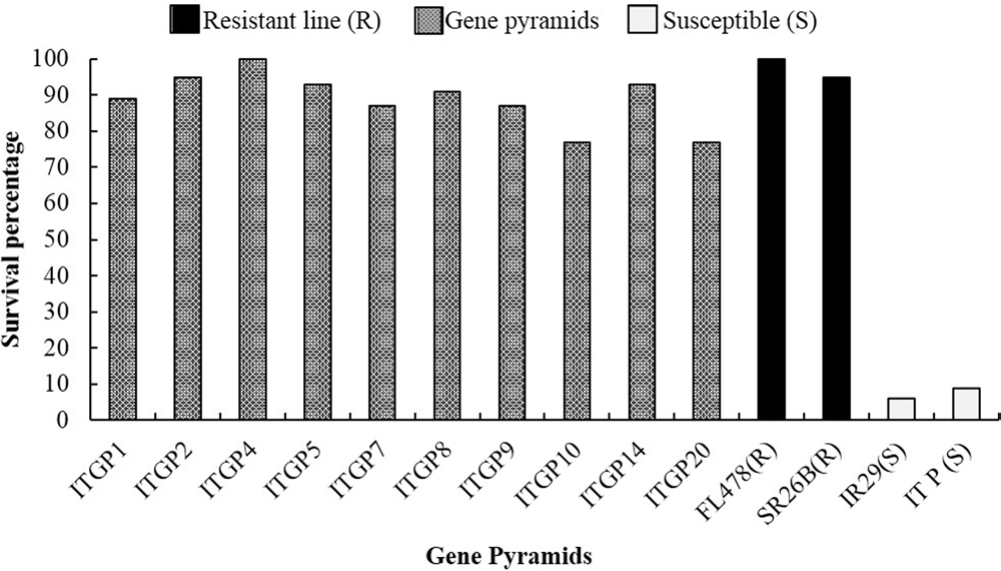
Survival rates of different gene pyramids after salinity stress.

For screening against salinity, FL478 (donor) and SR26B were employed as tolerant controls and IR29 and the recurrent parent as susceptible controls. All the seedling stage gene pyramids with *Saltol* QTL showed high degree of tolerance to salinity stress with most of them exhibiting >86% survival. The gene pyramid ITGP4, exhibited complete survival with a score 1, a reaction similar to FL478, the resistant control. Two gene pyramids showed less than 80% survival rate, while the susceptible controls, IR29 and Improved Tapaswini, showed only 6 and 8.8% survival respectively (Fig. 7). Our salinity bioassay result implied that the positive gene pyramids showed seedling stage salinity tolerance.

### Agronomic performance and grain quality

From the thirty two lines with different gene combinations identified, ten promising lines (Fig. 3), all four gene combinations, were selected and evaluated under field condition for their agronomic performance and produce was used for grain quality studies.

The results revealed that majority of the selections were close to the recurring parent (Table 3) with respect to yield components. Significant variation was not observed for traits like days to 50% flowering (except ITGP2, 20), plant height and number of ear bearing tillers (except ITGP10). The plant height of the Improved Tapaswini gene pyramids was in the range of 95 cm (ITGP4) to 104 cm (ITGP2) while the parental value was 99.0 cm. In case of panicle length, the range observed in the gene pyramids was 23.0 cm (ITGP1) to ITGP20 (29 cm) and the minimum value is closer to the parental value (23.5 cm). All the gene pyramids except ITGP1, had longer panicles than the parent. In general, the values of leaf traits i.e. length, width and the thousand grain weight are on the higher side in the gene pyramids. The line yields of all the gene pyramids were lower than the recurrent parent. The results on the grain quality suggest that some gene pyramids were better than the recurrent parent for important quality traits like kernel length and KLAC and all pyramid lines showed a desirable KALC value, which is identical to Improved Tapaswini. When the grain and quality traits were compared, five pyramids and the parent had similar HRR value. All gene pyramids had amylose content in the range of 25.04 to 22.79% (intermediate range) and the values are close to the value of Improved Tapaswini (24.52%) (Table 4).

**Table 3:** Morpho-agronomic characteristics of selected gene pyramids.

Pyramid line	DFF	PH	PL	LL	LW	EBT	GL	GW	1000 gw	LY
IT- P	102	99.00	23.50	36.50	1.07	13.0	7.47	2.58	19.06	4807
ITGP1	104	98.00	23.00	34.00	1.35	13.6	7.28	2.85	23.00	4314
ITGP2	115	104.0	25.00	41.00	1.06	11.3	7.68	2.57	25.22	4498
ITGP4	104	95.00	28.00	40.50	1.45	12.6	7.84	2.48	24.00	4330
ITGP5	106	101.0	25.00	40.50	1.07	12.0	7.89	2.78	24.30	4495
ITGP7	110	100.0	27.50	40.50	1.45	12.0	8.22	2.48	23.73	4195
ITGP8	109	99.50	24.00	37.50	1.40	12.0	7.20	2.45	22.50	4181
ITGP9	110	98.66	25.50	37.00	1.35	13.0	7.78	2.24	19.26	4470
ITGP10	110	99.50	27.00	39.50	1.30	9.60	7.17	2.36	22.10	3932
ITGP14	110	101.3	24.50	38.00	1.25	12.6	7.46	2.25	23.00	3645
ITGP20	115	97.50	29.00	37.00	1.05	10.0	7.45	2.56	20.62	4104

DFF- Days to fifty percent flowering; PH-Plant height; PL- Panicle length; LL- Leaf length; LW- Leaf width; EBT- Ear bearing tillers; GL-Grain length; GW- Grain width; % Fertility-Fertile spikelets/total spikelets (%); 1000 gw-grain weight of 1000 grains (gms); LY-Line yield (kg/ha).

**Table 4:** Grain quality traits of the selected gene pyramids.

Pyramid line	Hull	Mill	HRR	KL	KB	L/B	ASV	VER	KLAC	AC
IT-P	79.0	72.0	68.0	5.79	2.78	2.09	4	3.75	9.5	24.52
ITGP1	76.5	71.2	62.5	5.56	2.49	2.63	4	3.75	9.7	23.43
ITGP2	80.0	73.5	65.0	5.49	2.74	2.38	4	3.75	9.0	24.90
ITGP4	80.5	72.0	66.5	5.91	2.68	2.58	4	3.75	9.8	25.04
ITGP5	74.5	69.0	67.5	5.22	2.78	2.24	4	3.75	9.1	23.32
ITGP7	81.5	74.0	63.5	5.63	2.60	2.55	4	3.75	9.8	24.67
ITGP8	72.0	68.0	59.5	5.88	2.60	2.66	4	3.75	9.1	23.34
ITGP9	77.5	67.5	54.0	5.92	2.50	2.38	4	3.75	9.1	22.79
ITGP10	79.0	72.0	68.5	6.03	2.71	2.63	4	3.75	9.9	23.58
ITGP14	81.5	73.0	66.0	5.88	2.61	2.65	4	3.75	9.0	23.54
ITGP20	79.0	71.0	64.0	5.05	2.57	2.35	7	3.75	9.0	23.24

Hull-Hulling (%); Mill-milling (%); HRR-Head rice recovery (%); KL-kernel length (mm; KB-kernel breadth (mm), L/B-length breadth ratio; ASV-alkali spreading value; VER-volume expansion ratio; KLAC-kernel length after cooking; AC-Amylose content (%).

Of the 600 SSR markers screened, 44 markers showed polymorphism (Supplementary Table 1). From the 32 lines available at the BC_3_F_3_ generation, based on the SSR background selection, 10 gene pyramids having closer similarity to the recurrent parent were selected. In the ten gene pyramids and the recurrent parent, a total of 69 amplicons were amplified with the number of alleles were in the range of 1–3 and the mean PIC value of all markers was 0.20 with a highest value of 0.87 (RM5711) and a lowest value of 0.07 (RM1278) (Supplementary Table 1).

The gene combinations of the selected gene pyramids are: Four gene pyramids (ITGP1, ITGP2, ITGP4, ITGP5) with all ten target genes/QTLs (*Xa4* + *xa5* + *xa13* + *Xa21* + *Gm1* + *Gm4* + *Pi2* + *Pi9* + *Sub1* + *Saltol*), one (ITGP 7) with 8 genes/QTLs (*Xa4* + *xa5* + *xa13* + *Xa21* + *Gm1* + *Gm4* + *Sub1* + *Saltol*), one (ITGP 8) with 7 genes/QTLs (*Xa4* + *xa5* + *xa13* + *Xa21* + *Pi2* + *Sub1* + *Saltol*) and four (ITGP9, ITGP1, ITGP14, ITGP20) with 6 genes/QTLs (*Xa4* + *xa5* + *xa13* + *Xa21* + *Sub1* + *Saltol*).

### Background profiling with morphological and quality data

Using combined values of agronomic and quality traits, genetic distances between the 10 pyramid lines and the parent was calculated between each pair of observations. The average distance of recurring parent to all pyramid lines was 0.43. The result of pair wise comparisons indicated that ITGP 5 and ITGP10 were closer to IT parent with 0.28 distance. ITGP1, ITGP7 and ITGP4 were closer to parent with a distance of 0.34, 0.32 and 0.39 respectively while ITGP2, ITGP8, ITGP9 and ITGP20 were related to the parent with 0.53, 0.48, 0.47 and 0.49 distances respectively.

**Figure 8:**
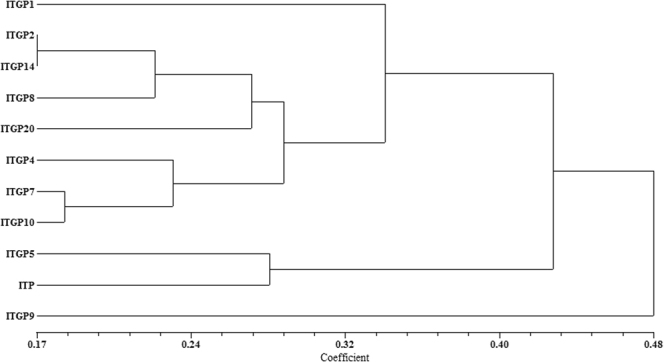
Dendrogram showing the genetic relationships between Improved Tapaswini (RP) and selected gene pyramids based on combined data of morphological and quality traits.

In the dendrogram generated based on a combination of morphological and quality traits, the eleven entries (10 pyramids + RP) were grouped into three major clusters with cluster I having only one entry (ITGP9). The cluster II consists of ITGP 5 and RP while cluster III was further divided into two sub clusters, Cluster III-A and III-B with Cluster III-A having ITGP 1 while cluster III-B had seven pyramids (Fig. 8). The analysis suggest that ITGP 5 was closer to the recurrent parent while all other pyramids except ITGP 9 are closer to the parent at various levels.

## Discussion

In the beginning of our study, clear polymorphism was observed with all the markers linked to the genes/QTLs (Table 1) between the Improved Tapaswini (recurrent parent) and the donors (Fig. 1). The novel feature of the study was the stacking and successful demonstration of the function of ten different genes/QTLs that govern the resistance/tolerance of five different stresses into an elite variety (Fig. 2) a first report in conventional breeding. Of these, two genes having broad spectrum resistance against blast (*Pi 9* from *O*. *minuta*) and bacterial blight (*Pi2* from *O*. *longistaminata*) had their origin from wild relatives. The two QTLs that confer tolerance to submergence (*Sub1*) and salinity (*Saltol*) are well known for their function. While the desired alleles of genes/QTLs was accomplished through effective foreground selection over ten generations using appropriate co-dominant markers, maximum recovery of the recurrent parental genome was attempted using both conventional (agronomic, grain quality) and using SSR markers. Out of the thirty two lines, ten selected promising lines with the presence and absence of targeted genes/QTLs with different gene combinations (Fig. 3), were evaluated in further studies.

Development of broad-spectrum resistance against bacterial blight in India is a great challenge due to the presence of a number of genetically distinct virulent Xoo strains in different geographical locations^13^. As probability of effectiveness of a combination of two or more genes is much higher than a single gene to defeat simultaneous pathogen mutations for virulence^58^, assemblage of several resistance genes into the host plant is a viable and practical strategy.

In the bioassays, all the gene pyramids, having all the BB resistance genes, displayed high levels of resistance against BB (Fig. 4) comparable to resistance shown by Improved Tapaswini, the recurrent parent. Three pyramids i.e. ITGP4, ITGP7 and ITGP8 displayed high levels of resistance with less than 0.9 cm lesion lengths, a result similar to Improved Tapaswini, the recurrent parent (Fig. 4) against BB and the results are in agreement with earlier reports^13–15,59–61^.

Though *Xa21* gene was reported to provide broad spectrum resistance against BB races that are spread over Africa and Asia^62,63^, in the study, selection was practiced to stack all the four genes (*Xa4*, *xa5*, *xa13* and *Xa21*) in the gene pyramids as the combination of *Xa4* + *xa 5* + *xa 13* + *Xa21* was superior to all other gene combinations of 1,2,3 genes^13,14,64^. In contrast, Tapaswini, the original parent of Improved Tapaswini, was highly susceptible to BB despite the presence of *Xa 4* gene suggesting its breakdown. The enhanced level of resistance conferred by more than one gene against a single pathogen has been described as quantitative complementation or synergistic action^65^.

Breakdown of resistance is well-known against blast disease and pyramiding of multiple resistance genes into a single genetic background is considered as a practical strategy to prevent/delay the breakdown of resistance of pathogens^66^. Since *Pi2* + *Pi9* combination was considered ideal, programs were taken up at CRRI to address the problem of blast in upland varieties^66–68^.

In the bioassays conducted at Central Rainfed Rice Research Station, Hazaribagh, Jharkhand, India, a hot spot for blast disease, ITGP2 and ITGP5 showed a highly resistant reaction with the score ‘0’, while ITGP1 and ITGP4, despite having *Pi2* + *Pi9*, showed moderately resistant reaction with the score ‘3’. The other line, ITGP8, having only *Pi2* also showed a moderately resistant (MR) reaction with the score ‘3’. The parent and the susceptible genotype showed a score of ‘4’ while both the donors (*Pi2* or *Pi9*) showed a highly resistant reaction. It was evident from the results that the gene combination *Pi2* + *Pi9* were more effective as they showed resistant reaction (Table 2). In the present study, two lines (ITGP1; ITGP4) showed a moderate reaction even with the two gene combination. This deviation could be a result of either admixtures or errors in sowing as these tests are conducted in screening nurseries at seedling stage and all the entries are surrounded by susceptible checks. This explanation is reasonable in the context as ITGP8, a line having only *Pi2* showed moderate reaction. Further studies are on to resolve the deviation exhibited by these two gene pyramids.

In the bioassays against gall midge, the gene pyramids, carrying both the gall midge resistance genes *Gm1* and *Gm4*, displayed complete resistance against gall midge. The gene pyramids such as ITGP2, ITGP4 and ITGP5 showed high degree of resistance with 80%, 90.9% and 86.6% positive plants respectively and in ITGP7, high degree of resistance was observed with 100% positive plants while in ITGP1, the survival was 66.6%. The other gene pyramids, without any of the gall midge resistance genes, as expected, showed complete susceptibility similar to recurrent parent and susceptible control T(N)1 (Fig. 5). The current result suggest the effectiveness of this gene combination against gall midge, a devastating insect pest on rice.

Ensuring food security in the context of climate change is the foremost challenge and improvement of rice varieties that can resist adverse conditions such as submergence and salinity is a priority. With a wide range of changes happening with El Nino effect, ensuring stable production and productivity of rice, the crop of millions of poor farmers of Asia, is a major concern as millions of hectares of monsoon based rice crop is severely affected by submergence or salinity or both, year after year. Though several genes and quantitative traits loci (QTL) linked with tolerance to salinity and submergence were reported, the most prominent and effective are *Sub 1* for submergence and *Saltol* for salinity. Till date, only FR13A, a submergence tolerant landrace has been extensively exploited in breeding^69^. FR13A, follows a quiescence strategy (i.e., the low-oxygen quiescence syndrome^70^) in which shoot elongation is suppressed to preserve carbohydrates for a long period (10–14 days) under flash-flood conditions and can restart its growth during de submergence by using preserved carbohydrates. The Sub1 QTL positioned on chromosome 9 rice was eminent as a leading gene conferring submergence tolerance in FR13A and with the development of Swarna Sub 1, through transfer of *Sub1* from FR13A into Swarna, the efficacy of the *Sub1* QTL was well established^71^. The effectiveness of *Sub1* QTL was also evident in the present study, as all the selected gene pyramids having *Sub1* QTL, displayed high degree of tolerance to submergence similar to FR13A with high survival rates [83.3% (ITGP1) to 100% (ITGP10)] (Fig. 6). Sub1BC2, an Indel marker, located between Sub1B and Sub1C genes, is closely linked with Sub1A gene, is being widely used as the marker for Sub 1A into elite cultivars^72^ and the variations observed in the gene pyramids in its expression needs further studies. However, with the use of gene specific markers like GNS2 and AEX, further improvements are possible.

Rice is reported to be sensitive at both vegetative and reproductive stages to salinity^73^ and Salt tolerance is a complex quantitative genetic character controlled by many genes^74,75^. Even though several genes are known to contribute salt tolerance, *Saltol*, a major QTL located on chromosome1, present in Pokkali^53^, confers tolerance in rice at vegetative stage through control of Na-K absorption ratio. Though Pokkali is the most widely used donor for salt tolerance, FL478, a derivative from IR29/Pokkali cross, has become a novel source of salinity tolerance because it possessed higher salinity tolerance than Pokkali and possessed many desirable traits like plant type, grain quality^76^.

All the seedling stage gene pyramids having *Saltol* QTL survived the saline stress showing varying degrees of tolerance. The gene pyramid ITGP4 showed complete survival after stress, a result similar to tolerant control (FL478) while other gene pyramids showed >86% (87–95%) survival except ITGP10 and ITGP20 which recorded 77% survival (Fig. 7). The varying degrees of tolerance expressed by different gene pyramids could be due the combination of different genes/QTLs (other than *Saltol*) that govern tolerance to salinity present in different pyramids. Further refinement of recombinants is in progress.

One of the observations in the study was the interaction between the stacked genes. Of the gene combinations possible with the four genes of BB, two combinations i.e. *Xa4* + *xa5* + *xa13* and *Xa4* + *xa5* + *Xa21* were not recovered and both the combinations involve *xa5*. Similar observation was made with the same genotype in our earlier study that is related to the development of Improved Tapaswini^13^. In our studies with IR 64, structural changes were observed in case of *Xa21* gene in gene pyramids (Das, Personal communication) while such structural changes were not recorded in the present study though large populations were screened in each generation. Reports of interaction between introgressed gene and reporter sequences having homology are available in transgenic rice^77^. The non-recovery of some combinations appears to be genotype specific as we could recover all the gene combinations with the same set of genes^14,78^ in our studies with other genotypes while non-recovery of some combinations with BB genes in a MAS based study was reported^79^ in addition to our reports with Tapaswini. Further studies are needed to draw definite conclusions on the gene-gene interactions. We also could not find any interaction between genes associated with resistance genes of blast and gall midge. Though *Pi2* and *Pi9* are located at the same locus on chromosome 6, both genes remained distinct with demonstrated independent function and this might be the reason for not observing any interaction between them. The probable reason for non-interaction between the GM genes which are located on different chromosomes, might be some structural dissimilarity. Of the two QTLs employed in the study, *Sub1* is known to be an ethylene response factor while *Saltol* is associated with Na-K absorption and due their differences in function, no interaction was observed.

The pyramiding of genes was accomplished using a systematic approach and at the end of the first cycle itself, six populations each having a different gene/QTL but similar to the recurrent parent in morphology and grain shape were generated. Practice of both foreground and background selection has resulted in identification of lines closer to the parent in morphology but with different combination of genes. The use of molecular markers (SSR) that were unlinked to the assembled genes/QTLs for background selection, has enhanced the proportion of recovery of the recipient genome. The high variance present at BC_1_F_1_ than those in subsequent generations, indicate a wider frequency distribution in the BC_1_F_1._ The desired genotype with recombination between the target gene locus and either one of the flanking markers is expected to occur at a much higher frequency in BC_1_F_1_ than BC_2_F_1_ suggesting the feasibility of practicing background selection in the BC_1_F_1_ generation. Thus, in addition to the background selection in the BC_3_F_1_, adding one round of background selection in BC_1_F_1_ to the MAS scheme may greatly increase the efficiency of the program and maximum recovery of recurrent parent genome was reported using SSR based background selection in the BC_1_F_1_ generation^80–82^. In the present study, morphology and grain quality based selection at each stage has greatly helped in recovery of the recurrent parent genome to the maximum extent despite the use of seven different parents and the back ground selection with SSR markers led to the selection of pyramids having closer similarity to the recurring parent (Improved Tapaswini), a result which is conformity with earlier reports which used varied markers like RFLP^79^, AFLP^81^ and SSR^13–15,30,36,83^ to achieve the same.

Furthermore in the present study, the Improved Tapaswini gene pyramids were evaluated for their closeness according to their agro-morphological and grain quality traits with the recurrent parent Improved Tapaswini. Combining both morphological and grain qualities the dendrogram showed that the selected ten gene pyramids were closest to the recurrent parent with very less distance. The average genetic distance of recurring parent to ten pyramid lines was less than 0.50 distance using combined values of agronomic and quality traits (Fig. 8). However, selection studies with more number of polymorphic SSR markers can clearly assess the proportion of the recurrent parent genome in these gene pyramids.

From the earlier study, with the mapping and identification of microsatellite markers like RM444 linked to *Gm1*, all the major gall midge resistance genes have tags, flooring the route for gene pyramiding of two or more genes into elite rice cultivars for durable resistance. *Gm1* was also introgressed into popular high yielding, rice cultivars Swarna and Samba Mahsuri, using the closest marker RM444 in a backcross breeding programme. It was also previously reported that, for disease resistance in *Gm4* region, RPP13- like protein were identified.The marker RM547 flank the *Gm4* gene at a distance of 1.9 cM. can be successfully used in MAB for development of gall midge resistant varieties^84–86^. As the same as previously reported we successfully transferred two gallmidge resistance genes *Gm1* and *Gm4* in Improved Tapaswini background using the flanking markers RM444 and RM547 respectively. Through map-based cloning approach the broad-spectrum rice blast resistance gene *Pi9* was cloned,which led to the identification of tandemly arranged six resistance-like genes with a NBS (nucleotide-binding site) and LRRs (leucine-rich repeats) in the *Pi9* locus. Both the rice *Pi2* and *Pi9* locus harbors multiple resistance (*R*) genes each controlling broad-spectrum resistance against diverse isolates of fungal pathogen, *Magnaporthe oryzae* causing devastating blast disease to rice. As per the previous report we succesfully transferred these two broad-spectrum rice blast resistance genes *Pi2* and *Pi9* in Improved Tapaswini background using the closest markers RG64 and P28 respectively^87,88^.

The molecular and physiological mechanisms involved in the submergence responses in plants have largely been characterized in two tolerant species, rice (*Oryza sativa*) and *Rumex palustris*. Rice SUB1A is an ethylene responsive factor (ERF) like transcriptional regulator, a member of the ERF gene family, dramatically improves submergence tolerance in controlled and field conditions. From the earlier report it was confirmed that SUB1A was highly expressed in FR13A and almost not detected in IR42. With reference to previous reports we have succesfully transferred the highly tolerant rice submergence QTL *Sub1* using FR13A, in Improved Tapaswini background using the closest markers SUB1BC2^89–91^. In a genome scale gene expression analysis it was found that between the two genotypes salinity suceptable IR29 and tolerant FL478 both are strikingly different at their transcriptional perspective, under salinity stress. Expression of IR29 was of relatively large number of genes as compared to the tolerant FL478. FL478, was responsible for maintaining low Na+, high K+, and Na+/K+ homeostasis in shoots of rice. Several salt tolerant rice lines has been developed by incorporating *Saltol* QTL into modern high yielding and salt-sensitive rice varieties through a targeted marker assisted backcrossing and marker assisted selection approach. In the previous study it was also further verified that, RM10745 is beneficial for marker-assisted selection of *Saltol* QTL^92,93^. Same as earlier study we have succesfully transferred the highly tolerant rice salnity QTL *Saltol* using FL478, in Improved Tapaswini background.

Four gene pyramids (ITGP1, ITGP2, ITGP4 and ITGP5) that have all the ten genes hold huge potential in rice breeding as they possess high levels of resistance/tolerance against multiple stresses. Though the integration and expression of ten or more genes using transgenic approach was demonstrated in rice^94^. The release of transgenic rice for general cultivation remains uncertain unless the issues related to human biosafety and transgenics are resolved. After their release, the MAS products can address both the biotic and abiotic stress simultaneously and having morphology and grain quality similar to the parent, the farmers with their popular variety can realize significant yield grains thereby enhancing their farm income and the variety with its stable yields, can lead to enhanced rice production in eastern India.

The rising number of pathogenic variants of different diseases and appearance of new and active insect biotypes, necessitates immediate attention. Through conventional breeding, pooling of ten different genes/QTLs will be a herculean task and the advances in marker technology has provided us a great opportunity to achieve the precise transfer and stacking of ten genes/QTLs with significant savings in time, space, labour and money. In addition, the end products can reach farmers directly as they need not pass thorough any regulatory approvals. Though the yield levels of the gene pyramids is lower than the recurrent parent, large scale field trials are needed to confirm the observation and if any yield penalty exists, detailed investigations can dissect the influence of individual gene(s)/QTL(s), gene combinations/interactions on yield and yield attributes using the large number of gene pyramids developed in the study. This novel demonstration of pyramiding ten (six + existed four) different genes/QTLs and their expression at desired levels in a new genetic background can herald a new revolution in molecular plant breeding. This achievement is a clear demonstration of the immense potential of modern plant breeding to confront several biotic and abiotic stresses at the same time and can greatly help in increasing both production and productivity of rice in the years to come.

## Materials and Methods

### Plant material and breeding scheme

The recurrent parent employed in the study was Improved Tapaswini, a gene pyramid of Tapaswini, developed at Central Rice Research Institute, Cuttack^68^. Tapaswini (CR 333-6-4), is a derivative of Jagannath/Mahsuri cross combination, is a highly popular *indica* cultivar with the rice farmers of Orissa in 1996 because of its high yield, medium slender grains and good cooking quality and can be grown in both the seasons. The variety was initially released for cultivation in Orissa, an Eastern state in India, and now its cultivation has now spread to five more eastern states.

Though high in popularity, it is susceptible to bacterial blight and several other stresses. To address the problem, in an earlier study, four bacterial blight resistance genes (*Xa4*, *xa5*, *xa13* and *Xa21*) were introduced into Tapaswini using MAS approach and NILs having desired levels of resistance to BB were developed and one of the NILs [CRMAS 2622-7-6 (IET 21070)] was released as Improved Tapaswini for cultivation in BB endemic areas. The Improved Tapaswini did show higher levels of resistance against BB and did not record any yield penalty in the two year mandatory multi-location trials (10 and 18 locations) conducted by AICRIP, the nodal agency in India^95^. The new cultivar has been accepted by the farmers as it closely resembles the parent in both morphology, yield and grain and cooking quality but with enhanced resistance to BB. But, the variety despite its popularity is susceptible to pathogens like blast and gall midge and environmental stresses like submergence and salinity.

The donors used in the study include two breeding lines WHD-1S-75-1-127 (*O*. *minuta* derivative-*Pi9*)^66^ and C1O1A51(*Pi2*)^96^, for blast resistance, two released varieties Kavya (*Gm1*)^97^, a derivative of the cross WGL 27120/WGL 7672//Mahsuri/Surekha, and Abhaya(*Gm4*)^34^ are resistant to different gall midge biotypes, the popular FR13A^98^ for submergence tolerance QTL (*Sub1*) and a breeding line FL478, for the salinity tolerance QTL (*Saltol*)^53^. FL478, a breeding line from IRRI, Philippines, contains a 1 Mb DNA fragment (10.6–11.5 Mb on chromosome1) that contains the entire *Saltol* locus of Pokkali, a well-known donor for salinity tolerance, and the fragment was flanked by IR29 alleles^54^ (Kim *et al*. 2009). The primers employed for all the ten target genes/QTLs were all from published reports (Table 1). Due to its popularity and farmers preference, the Institute (CRRI) has initiated a program to enhance the defence capabilities of improved Tapaswini further through transfer of most effective gene (s)/QTL(s) to address submergence (*Sub1*) and salinity (*Saltol*) or gene combinations to address blast (*Pi2* + *Pi9*), gall midge (*Gm1* + *Gm4*).

### Parental polymorphism survey

For the parental polymorphism survey, all the parents involved were included i.e. Improved Tapaswini, the recipient parent and donors: Kavya (*Gm1*-gall midge), Abhaya (*Gm4*-gall midge), C1O1A51 (Pi2-blast), WHD-1S-75-1-127 (*Pi9*-blast), FR13A (*Sub1* QTL-submergence) and FL478 (*Saltol* QTL-salinity). For the survey, markers linked to different genes/QTLs like RM444 (*Gm1*), RM547 (*Gm4*), RG64 (*Pi2*), P28 (*Pi9*), SUB1BC2 (*Sub1*) and RM10745 (*Saltol*) were employed to examine the relation between the markers and the resistance/tolerance and susceptible nature of the parents for each trait under study.

### DNA isolation and PCR analysis

Mini scale DNA isolation for PCR analysis was carried out as per Dellaporta *et al*.^99^. As stated earlier, the primers employed for the target genes/QTLs were all from published reports^53,84,100–106^. The reaction mixture for PCR analysis contained 50 ng of DNA template, 5 pico M of each of the primers forward and reverse, 200 μM dNTPs, 1× PCR buffer (10 mM Tris-HCl, pH 8.3, 50 mM KCl, 1.5 mM of MgCl_2_ and 0.01 mg/ml gelatin) and 0.5 U of Taq DNA polymerase (DreamTaq DNA Polymerase, Thermo Scientific) in a quantity of 20 µl. All the PCR assays were performed as described earlier for each marker and the separated PCR products were visualized under UV light and photographed using an alpha imager (Alpha Innotech, USA). The PCR products of RG64 (*Pi2*) and RG556 (*xa5*) were digested with restriction enzymes *Hae III* and *DraI* respectively as per manufacturer’s instructions. The products amplified by RG 64 and RG 556 primers were monomorphic, hence restriction digestion was carried out and the digested products did show polymorphism associated with resistance and susceptibility.

### Bioassays against bacterial blight

For screening of Tapaswini gene pyramids against the bacterial blight pathogen (BB), the inoculum of BB isolates recommended at CRRI was used. The inoculum was prepared by suspending the bacterial mass in sterile water to concentration of approximately 10^9^ cells/ml. The upper three leaves of the plants were cut and clip-inoculated with the bacterial suspension at the maximum tillering stage as per^107^. The observations were recorded based on visual scoring and lesion length (LL) measurement 20 days later. The threshold level employed for distinguishing resistant and susceptible reaction based on lesion length was <5 cm (resistant) and >5 cm (susceptible).

### Bioassays against blast

The screening was carried in the Uniform Blast Nursery (UBN) pattern, where each test entry (gene pyramids, parent, donor, susceptible controls) was sown as a single line with a 10 cm distance between consecutive rows and the susceptible control (B 40) are placed at the end of 20 rows of test entries. All the rows in the nursery were surrounded all around by two rows of HR12, a well-known blast susceptible line to spread the disease. High dosage (120 kg N/ha) of nitrogen was applied to the nursery for promotion of infection. Supplementary inoculums, in the form of chopped diseased leaves were scattered over the nursery and transplanting of infected seedlings in the midst of border rows. The scoring on SES scale^108^ was recorded at 10 days intervals starting from 25 to 30 days, depending to the severity of leaf blast and at least two readings of blast score were recorded. The bioassay was carried out with three replications with 18 plants constituting each replication.

### Bioassays against gall midge

Ten days old seedlings of selected pyramids, donor parents (Kavya, Abhaya) and a susceptible control [T(N)1] were planted in plastic trays and the bioassays were conducted as per standard protocol^109^. The trays were kept in cages and gall midge larvae were released on the seedlings inside the cages and the reaction scores like seedling damage (in %), emergence and frequency of silver shoot (galls) were recorded after twenty days. The classification of lines into resistant and susceptible classes was recorded when the susceptible plants showed 90–100% plant damage with the emergence of galls. The plants free of galls were cut to observe the dead maggots and tissue necrosis to confirm the resistance status. The threshold level employed for distinguishing resistant and susceptible reaction based on injury (%) was 0–20 percent (resistant) and >20 percent (susceptible). The bioassay was carried out with three replications with 18 plants constituting each replication.

### Screening for submergence tolerance

Three week old seedlings of gene pyramids, Improved Tapaswini (recipient parent) and controls FR13A (resistant) and IR42 (susceptible), raised in plastic trays, were subjected to submergence stress by placing the trays in submergence tanks of CRRI. The tanks were filled with water slowly and the plants were subjected to stress of total submergence by keeping 1.8 m standing water in the tanks. At the end of 15 days, the tanks were drained and the survival rates were recorded. The trays with the stressed plants were kept in open for desubmergence and the survival rates were recorded again after 8 days of desubmergence^110^. The screening was carried out with three replications with 18 plants constituting each replication.

### Screening for salinity tolerance

Three week old seedlings of gene pyramids were transplanted along with Improved Tapaswini (recipient parent) and controls i.e. FL478 (resistant), SR26B (resistant) and IR29 (susceptible) and the screening was conducted as per Fageria^111^. After two days, the experimental tanks were filled with salt water and a stress of 8–10 dS m^−1^ EC was maintained for ten days and the stress was increased to 16 dS m^−1^ and at the end of 60 days (when the susceptible controls are completely dead), the survival rates and data on other parameters was recorded. The experiment continued with the surviving lines till maturity and spikelet fertility (%) was recorded. The screening was carried out with three replications with 18 plants constituting each replication.

### Characterization for morphological and quality traits

Thirty-day-old seedlings of the recurrent parent and selected gene pyramids were transplanted with 15 × 20 cm spacing in a randomized complete block design with three replications at the experimental farm of Central Rice Research Institute (CRRI) Cuttack. Data was recorded on five plants from each line for agronomic traits, namely DFF (days to 50% flowering), plant height, tillers/plant, panicle length, number of filled grains/panicle and 1000-grain weight and line yields were calculated and extrapolated to kg/ha.

Characterization of grain and cooking quality traits was performed on the grains harvested from BC_3_F_3_ generation. For physical characters, data was collected from ten fully developed milled rice kernels. The kernel elongation ratio (KER) was expressed as the ratio of the average length of the cooked kernels to that of the uncooked kernels. The average ratio of the length of cooked rice to kernel length of milled rice of ten kernels was elongation ratio (VER). For calculating volume expansion, the standard method^112^ was followed. By the alkali digestion test^113^, alkali spreading value (ASV), gelatinization temperature was estimated indirectly. Following the method described by Juliano^114^, amylose content was determined.

### Statistical analyses

Amplified products were scored for presence (1) or absence (0) for each primer genotype combination and the PIC value of a marker was calculated according to^94^. Dice similarity co-efficient was computed and used for generating a dendrogram following UPGMA. The computer package NTSYS-PC-2.02f^115^ was used for cluster analysis.

## Conclusion

Improvement of high degree of resistance against diseases (BB and blast) and insects (gall midge) in the Indian subcontinent is a leading challenge because of high diversity of agro-climatic zones that conferring tolerance to submergence and salinity. The two major abiotic stresses is critical in the climate change scenario. The current study, presents the gene pyramiding of six tolerance /resistance, genes/QTLs such as submergence (*Sub1*), salinity (*Saltol*), and resistance to blast (*Pi2*, *Pi9*), gall midge (*Gm1*, *Gm4*) into an elite cultivar, Improved Tapaswini, which already have four BB resistance genes (*Xa4*, *xa5*, *xa13*, *Xa21*). Out of a large BC_3_F_2_ population we obtained 250 lines, with resistance alleles in homozygous state. Afterward in BC_3_F_3_ generation we carefully selected 32 lines with diverse gene combinations. Finally we selected 10 promising lines having closer similarity to the recurrent parent, based on the SSR background selection with diverse gene combinations, for further refinement of these gene pyramids. The recurrent parent along with the pyramided lines showed high levels of resistance against the bacterial blight bioassay. Against gall midge, blast, submergence and salinity (seedling stage) stresses the recurrent parent showed complete susceptibility while the pyramided lines showed different percentages of resistance/tolorence. Among the 4 pyramided lines with ten genes, the ITGP2 and ITGP5 showed high degree of resistance to all the biotic stresses and tolerance to the abiotic stresses, while ITGP4 showed highest degree of resistance to all targeted stresses and moderate resistance to blast. The line ITGP1 showed resistance to all targeted stresses, but moderate resistance to blast. The other six gene pyramids in different gene combinations also showed high degree of tolerance/resistance to each specific stress. In case of the background selection using morphological and grain quality traits we obtained all the ten gene pyramids with more than 90% recovery of recurrent parental genome. Through utilization of phenotypic selection tied with marker-assisted selection, out of 10 gene pyramids first time we obtained 4 gene pyramids with desired 6 abiotic and biotic tolerance/resistance (*Sub1*, *Saltol*, *Pi2*, *Pi9*, *Gm1* and *Gm4*) genes/QTLs along with 4 bacterial blight resistance genes (*Xa4*, *xa5*, *xa13*, *Xa21*) in Improved Tapaswini background. Among the 4 pyramided lines with ten genes, ITGP2 and ITGP5 were obtained as best lines with high degree of resistance/tolerance to all the targeted biotic and abiotic stresses with maximum recovery of recurrent parental genome. The success of the study clearly demonstrates the efficacy of the marker technology in stacking of several agronomically important genes/QTLs into an elite genetic background and the tests on gene pyramids suggest that incorporation of strong and wide-ranging resistance/tolerance against many stresses at the same time is feasible. The other selected gene pyramids (having combinations of fewer genes <10), showing strong resistance/tolerance against each individual stress can be employed to address specific stress(s) present in different regions of India. The future prospect of the study intended on these gene pyramids, comprises a further background selection with additional numbers of high density markers and a multi-location trial against each targeted trait(s) to confirm the expression of the stacked genes/QTLs before their deployment. The achievement may possibly also encourage, several such studies to understand the prospective of molecular plant breeding as the basis for crop enhancement.

## Electronic supplementary material

Supplementary Table 1
