# Supplementary material for: Improved Tapaswini having four BB resistance genes pyramided with six genes/QTLs, resistance/tolerance to biotic and abiotic stresses in rice
Source: Sci Rep. 2018 Feb 5;8:2413. doi: 10.1038/s41598-018-20495-x (PMC5799378; doi:10.1038/s41598-018-20495-x)
Supplement: Supplementary file 1 — Supplementary Table 1 [file 41598_2018_20495_MOESM1_ESM.doc]

**Improved Tapaswini having four BB resistance genes pyramided with six genes/QTLs, resistance/tolerance to biotic and abiotic stresses in rice**

**Gitishree Das^1, 2*^, Gundimeda J. N. Rao^1,3*^, M. Varier^4^, A. Prakash^5^ & Dokku Prasad^1,6^**

^1^Crop Improvement Division, ICAR-National Rice Research Institute, Cuttack, Odisha, 753006, India.

^2^ Research Institute of Biotechnology & Medical Converged Science, Dongguk University-Seoul, Ilsandong-gu, Gyeonggi-do 10326, Republic of Korea.

^3^Department of Bio Sciences and Bio Engineering, Indian Institute of Technology Guwahati, Guwahati, 781039, Assam, India

^4^NRRI-Central Rainfed Upland Rice Research Station, Hazaribagh, Jharkhand, 825301, India.

^5^Crop Protection Division, ICAR-National Rice Research Institute, Cuttack, Odisha, 753006, India.

^6^ Kaveri Seeds, Secunderabad, Telangana, 500003, India.

Supplementary Table 1: The number of alleles detected in the gene pyramids with the SSR markers.

| **Sl. No.** | **Ch. No.** | **Locus** | **No of bands** | **PIC Value** | **Sl.No.** | **Ch.no.** | **Locus** | **No of bands** | **PIC Value** |
| --- | --- | --- | --- | --- | --- | --- | --- | --- | --- |
| 1 | 1 | RM212 | 2 | 0.14 | 23 | 9 | RM6839 | 3 | 0.00 |
| 2 | 3 | RM565 | 2 | 0.00 | 24 | 7 | RM5436 | 1 | 0.00 |
| 3 | 3 | RM232 | 1 | 0.00 | 25 | 8 | RM223 | 2 | 0.00 |
| 4 | 2 | RM138 | 1 | 0.00 | 26 | 1 | RM472 | 1 | 0.00 |
| 5 | 4 | RM3337 | 2 | 0.00 | 27 | 6 | RM587 | 2 | 0.66 |
| 6 | 4 | RM1155 | 2 | 0.76 | 28 | 6 | RM314 | 2 | 0.00 |
| 7 | 4 | RM1359 | 1 | 0.10 | 29 | 6 | RM584 | 1 | 0.00 |
| 8 | 3 | RM1278 | 3 | 0.07 | 30 | 9 | RM219 | 2 | 0.76 |
| 9 | 1 | RM3642 | 2 | 0.60 | 31 | 2 | RM279 | 1 | 0.00 |
| 10 | 1 | RM1201 | 1 | 0.00 | 32 | 11 | RM287 | 1 | 0.00 |
| 11 | 2 | RM233 | 1 | 0.00 | 33 | 2 | RM423 | 2 | 0.10 |
| 12 | 4 | RM3524 | 2 | 0.72 | 34 | 2 | RM324 | 1 | 0.00 |
| 13 | 7 | RM3583 | 1 | 0.00 | 35 | 6 | RM190 | 1 | 0.00 |
| 14 | 5 | RM3381 | 1 | 0.00 | 36 | 2 | RM475 | 1 | 0.19 |
| 15 | 5 | RM1248 | 2 | 0.66 | 37 | 2 | RM236 | 1 | 0.00 |
| 16 | 7 | RM5711 | 3 | 0.87 | 38 | 3 | RM442 | 1 | 0.00 |
| 17 | 5 | RM4838 | 2 | 0.58 | 39 | 6 | RM435 | 1 | 0.00 |
| 18 | 7 | RM3859 | 2 | 0.76 | 40 | 2 | RM482 | 1 | 0.00 |
| 19 | 1 | RM490 | 1 | 0.00 | 41 | 2 | RM521 | 1 | 0.00 |
| 20 | 1 | RM495 | 1 | 0.00 | 42 | 1 | RM246 | 3 | 0.00 |
| 21 | 4 | RM401 | 2 | 0.66 | 43 | 3 | RM570 | 1 | 0.00 |
| 22 | 9 | RM2144 | 2 | 0.50 | 44 | 7 | RM432 | 2 | 0.71 |
